# Reactants, products, and transition states of elementary chemical reactions based on quantum chemistry

**DOI:** 10.1038/s41597-020-0460-4

**Published:** 2020-05-08

**Authors:** Colin A. Grambow, Lagnajit Pattanaik, William H. Green

**Affiliations:** Department of Chemical Engineering, Massachusetts Institute of Technology, 77 Massachusetts Ave, Cambridge, MA 02139 United States

**Keywords:** Quantum chemistry, Cheminformatics, Reaction kinetics and dynamics

## Abstract

Reaction times, activation energies, branching ratios, yields, and many other quantitative attributes are important for precise organic syntheses and generating detailed reaction mechanisms. Often, it would be useful to be able to classify proposed reactions as fast or slow. However, quantitative chemical reaction data, especially for atom-mapped reactions, are difficult to find in existing databases. Therefore, we used automated potential energy surface exploration to generate 12,000 organic reactions involving H, C, N, and O atoms calculated at the *ω*B97X-D3/def2-TZVP quantum chemistry level. We report the results of geometry optimizations and frequency calculations for reactants, products, and transition states of all reactions. Additionally, we extracted atom-mapped reaction SMILES, activation energies, and enthalpies of reaction. We believe that this data will accelerate progress in automated methods for organic synthesis and reaction mechanism generation—for example, by enabling the development of novel machine learning models for quantitative reaction prediction.

## Abstract (summary)

**Table:** 

Measurement(s)	activation energy • Standard Transformed Enthalpy Change • transition state • reactant • reaction product • chemical reaction • SMILES string
Technology Type(s)	quantum chemistry computational method

Machine-accessible metadata file describing the reported data: 10.6084/m9.figshare.12047193

## Background & Summary

Rapid advancements in computational methods for chemical synthesis planning and automated reaction mechanism generation, especially in the area of machine learning, are causing a significant shift in how such problems are tackled. Deep learning approaches are replacing conventional quantitative structure-activity relationships often based on support vector machines, decision trees, or linear methods like partial least squares^1,2^. These new systems are becoming widely available for computer-aided retrosynthesis^3^, reaction outcome prediction^3^, high-throughput virtual screening^4^, and more general molecular property prediction^5,6^. Computational approaches are also increasingly common in reaction mechanism generation due to the large number of species and reactions that are generally required for accurate descriptions of phenomena like pyrolysis, combustion, and atmospheric oxidation^7–9^. Frequently, this involves characterizing chemical pathways with quantum chemistry^8^, but deep learning methods have also recently been applied to estimate thermochemistry during mechanism generation^10,11^.

While computers already outperform humans at qualitatively predicting reaction products^12,13^ and successful yield predictions have been demonstrated for limited datasets^14,15^, quantitative reaction information is still elusive in large databases like Reaxys^16^, Pistachio^17^, and the United States Patent and Trademark Office database^18^. Reaction yield, time, and some quantitative conditions like temperature are sometimes available, but there is usually no information on reaction kinetics. If such data were available, calculation of derived properties—such as minimum reaction times and branching ratios—would be possible. Our goal is to provide a quantitative dataset of reactions that enables the calculation of such data and can lead to more efficient drug design and help in deciding which reactions are important in mechanism generation.

Computationally generating a dataset of reactions is significantly more complex than only calculating stable equilibrium structures because transition states (TSs) of chemical reactions cannot be enumerated in the same manner as stable molecules. Even if the reactant and product structures are known, the exact TS geometry has to be found via a human-guided search or with expensive automated TS finding methods. Here, we use automated potential energy surface exploration to generate the dataset of reactions, which has been shown to be successful in cases when many reaction pathways have to be evaluated^19–21^. More specifically, we rely on the growing string method^22^ to automatically optimize reaction paths and TSs.

**Fig. 1:**
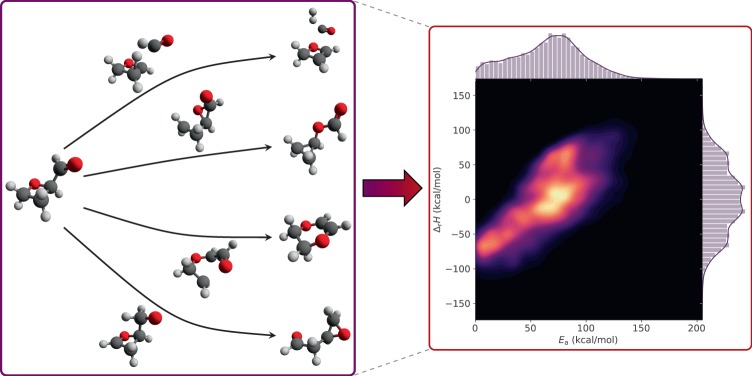
Reaction data generation and visualization of reaction space. During data generation, many reactants are optimized, hundreds of reaction paths for each reactant are searched with an automated transition state finding method, and the resulting products are optimized. The reaction space spans a wide range of activation energies and is visualized with a bivariate kernel density estimate (using a Gaussian kernel) of the probability density of the activation energy and enthalpy of reaction. The visualization encompasses both forward and reverse reactions.

We report quantum chemical data on more than 16,000 reactions in the form of reactants, products, and TSs at the B97-D3/def2-mSVP level of theory and 12,000 reactions at the *ω*B97X-D3/def2-TZVP level of theory. The data include the raw output from geometry optimizations and frequency calculations in addition to atom-mapped SMILES, activation energies, and enthalpies of reaction. All reactions are gas-phase calculations involving up to seven carbon, oxygen, or nitrogen atoms per molecule. The reactants are sampled from GDB-7, a subset of GDB-17^23^, meaning that all reactions have a unimolecular reactant but potentially multi-molecular products. Figure 1 illustrates the dataset generation process and the resulting space of reactions in terms of their activation energies and enthalpies of reaction.

## Methods

### Overview

The dataset generation procedure started by selecting molecules from GDB-7^23^, generating conformers, and optimizing the lowest-energy conformer. An exhaustive set of driving coordinates subject to valence and connectivity constraints were generated for each reaction. Reaction paths were calculated with the growing string method^22^, which searched along each of the driving coordinates. Products and TSs discovered in this way were reoptimized, duplicate reactions were removed, and checks were performed to verify the reactions. The generated reactions were then refined at a higher level of theory. Because of the large number of density functional theory (DFT) calculations required, the massively parallel nature of the calculations was exploited by running thousands of calculations in parallel on a supercomputer.

### Reactant optimization

Because of the unfavorable scaling of quantum chemical calculations, we only considered molecules with at most seven heavy atoms (C, N, O). *All* molecules with six or fewer heavy atoms were selected from GDB-7 (~770) and a random selection of ~430 molecules were selected from the set with seven heavy atoms. Starting from the SMILES strings, we embedded several hundred conformers for each molecule using the RDKit^24^ with the ETKDG distance geometry method^25^ and relaxed their geometries using the MMFF94 force field implemented in RDKit. The lowest energy structure was selected for each molecule and optimized at both the B97-D3/def2-mSVP with Becke-Johnson damping level of theory^26^ and the *ω*B97X-D3/def2-TZVP^27^ level of theory with Q-Chem 5.1^28^. We ascertained that none of the molecules contained imaginary frequencies. All calculations, including the subsequent string method calculations, were done in the singlet state and used a spin-unrestricted ansatz because the bond distortions occurring in the corresponding TSs might be better treated with an unrestricted formulation. The def2-mSVP basis set in the Karlsruhe *def2* basis set family^29^ is a modified version of def2-SV(P), which corrects for an overestimation of bond lengths involving hydrogen^30^. All DFT calculations used the *SG-2* standard quadrature grid, which is of sufficient quality for B97-based functionals^31^.

### Potential energy surface exploration

The most demanding and most time-intensive step of the reaction generation process is the optimization of reaction paths to the minimum energy paths (MEPs) containing the correct TS structures. We accomplished this in an automated fashion by using the single-ended growing string method (GSM)^22^ at the B97-D3/def2-mSVP level of theory. GSM performs the reaction path optimization using a set of delocalized internal coordinates, which means that the resulting MEPs may be slightly different than those obtained via a reaction path following procedure in mass-weighted internal coordinates^32^. Single-ended methods only require a reactant structure to find reactions whereas double-ended methods additionally require knowledge of the product^33,34^. *A priori* specification of the product can be problematic when there is no simple elementary step connecting reactant and product. Single-ended GSM solves this issue by only requiring a set of driving coordinates to initiate the reaction path search.

In our case, the driving coordinates are specified as bond transformations in terms of primitive internal coordinates. The direction given by the primitive internal coordinate vector is projected onto the nonredundant delocalized internal coordinates^35^, which is the space in which the reaction path optimization occurs. This results in a single tangent vector that represents all of the driving coordinates simultaneously. Importantly, this allows all other coordinates to change without constraint during the optimization, thus allowing necessary angle, torsion, and even additional bond changes to occur. Once a path has been grown, the entire path is optimized towards the MEP while monitoring the number of TSs along the path and truncating it if more than one TS is detected—ensuring that the reaction is elementary. As a result of this, not all bond changes given in the driving coordinates are guaranteed to occur. Towards the end of the path optimization, an exact TS search takes place guided by curvature information from the string.

In order to obtain many reactions, we generated an exhaustive list of driving coordinate sets for each reactant subject to a few constraints. Because elementary reactions usually involve few bond changes, we specified that at most two bonds could be broken, at most two bonds could be formed, and a total of at most three bonds could be changed. A “bond” in this sense ignored bond orders and only considered whether two atoms were connected to each other. Note that these constraints were only selected to ensure a computationally tractable number of driving coordinates. As described in the previous paragraph, these limits did not apply during the actual path optimization, they were only used to specify the initial search direction. We also ignored driving coordinates involving only a single bond change as these would likely correspond to barrierless associations or dissociations. Driving coordinates involving equivalent hydrogens were not included. Equivalent hydrogens only differ in their atom indices, *e.g*., a hydrogen atom that is part of a methyl group is considered to be equivalent to another hydrogen in the same methyl group. Lastly, the driving coordinates were further limited based on the valences of the expected product structures. Hydrogen atoms must have one bond, carbons can be connected to a minimum of two and a maximum of four atoms, oxygen to a minimum of one and a maximum of two, and nitrogen to a minimum of one and a maximum of three.

This process usually resulted in several hundred sets of driving coordinates per reactant. Following each GSM calculation, the endpoint of the paths were subjected to additional geometry optimizations to ensure that the product structures were at a minimum. For each reactant, there were many duplicate reactions. Instead of discarding all of them, up to four duplicates of the same reaction were retained for additional TS optimization in case some of the optimizations fail. While GSM already produces a mostly optimized TS structure, the additional optimization step ensured that the TSs were optimized to high accuracy.

### Reaction verification and extraction

After the additional TS optimizations, duplicate reactions were filtered out again. If duplicates were present, the lowest-barrier reaction was retained. Differences in barrier height may arise due to different TS conformers. Although GSM provided an optimized MEP for each reaction, it is possible that some reactions containing incorrect transition states remained. These were filtered out according to a normal mode analysis described in the Technical Validation section.

To convert from three-dimensional geometries to SMILES^36^, connections and bond orders could be perceived with Open Babel^37^. However, there were cases where the derived bond orders were chemically unreasonable, for example, when the resulting SMILES contained adjacent radical atoms which most likely correspond to double bonds. To eliminate unreasonable structures, we converted the Open Babel molecule to InChI^38^, which only treats bond orders implicitly and resolves the issue. A downside to using InChI is that tautomers are assigned the same string, but this can be circumvented by converting to a nonstandard InChI containing a fixed-hydrogen layer. Additionally, atom ordering was lost in the InChI conversion. We reconstructed the atom map by converting to an RDKit molecule and determining the graph isomorphism between the original molecule and the RDKit molecule without considering bond orders. In the future, an alternative procedure for perceiving SMILES could be implemented based on natural bond orbital analysis^39^.

The activation energies were extracted by adding the zero-point energies from a harmonic vibrational analysis to reactant, product, and TS energies and computing the difference between resulting TS and reactant energies. Similarly, enthalpies of formation were determined based on the difference of product and reactant energies.

### Refinement

B97-D3/def2-mSVP strikes a reasonable balance between cost and accuracy for potential energy surface exploration, but does not provide particularly accurate energies. Therefore, we refined the discovered pathways using *ω*B97X-D3/def2-TZVP. As mentioned earlier, reactants were already optimized with *ω*B97X-D3/def2-TZVP. Reactions were extracted as described in the preceding subsection, but some duplicates were retained to increase the probability of successful reoptimization. Only the duplicate with the smallest activation energy was retained in the end. Products and TSs were then reoptimized with *ω*B97X-D3/def2-TZVP and the final high-level reactions were extracted as before.

## Data Records

Q-Chem output files, extracted SMILES, activation energies, and enthalpies of formation are available for 16,365 B97-D3/def2-mSVP reactions and for 11,961 *ω*B97X-D3/def2-TZVP reactions^40^. The raw log files are stored in two compressed archive files, b97d3.tar.gz and wb97xd3.tar.gz for B97-D3/def2-mSVP and *ω*B97X-D3/def2-TZVP data, respectively. Each archive contains a separate folder for each reaction labelled rxn######, where ###### denotes the reaction number padded with zeros. Within each folder are the three log files for a reaction, r######.log for the reactant, p######.log for the product, and ts######.log for the transition state. Each log file contains the output of a geometry optimization and harmonic vibrational analysis.

Atom-mapped SMILES, activation energies, and enthalpies of formation for each reaction are listed in the comma-separated values files b97d3.csv and wb97xd3.csv for the B97-D3/def2-mSVP and *ω*B97X-D3/def2-TZVP levels of theory, respectively. The reactions are listed in the same order as the corresponding folders in the archive files. The columns in the comma-separated values files are explained in Table 1.

**Table 1:** A description of the columns in the comma-separated values files.

Column label	Description
idx	Reaction index
rsmi	Reactant SMILES
psmi	Product SMILES
ea	Activation energy (kcal mol^−1^)
dh	Enthalpy of reaction (kcal mol^−1^)

During the potential energy surface exploration, many duplicate reactions were encountered which were filtered out. Additionally, reactions that did not pass the tests described in the Technical Validation section were removed from the final list. Nonetheless, all of these calculations also produced optimized transition states, although the reactants and products were not verified for many of them, and duplicate transition states exist. These data may still prove to be useful if only transition state structures are required or if additional calculations are done to obtain the corresponding reactants and products. Therefore, the log files for all successfully optimized transition states at both levels of theory are stored in ts_with_dup_b97d3.tar.gz and ts_with_dup_wb97xd3.tar.gz. There are 69,366 B97-D3/def2-mSVP transition states and 24,987 $\omega$B97X-D3/def2-TZVP transition states.

## Technical Validation

Although the growing string method produces an optimized minimum energy path that should contain the correct TS in most cases, insufficient path discretization and reoptimization of TS geometries can lead to convergence failures or result in incorrect transition states. We performed several checks to filter out incorrect reactions. We ensured that all TSs have exactly one imaginary frequency. Reactions were also removed if the energy during the TS optimization changed by more than 3 kcal mol^−1^ relative to the highest energy on the growing string path. The most important check that we performed was to verify that the atomic displacements for the imaginary frequency matched the bond changes that occurred going from the proposed reactant to product. For each proposed reaction, we determined which bonds were changing in the reaction and ensured that the imaginary frequency normal mode displacements along those bonds were larger than the displacements along all the other bonds. This indicated that movement along the reaction coordinate mostly involved atoms undergoing significant change in the reaction. After all these changes, there is still the possibility that some of the transition states are incorrect. As a final check, we removed all of the reactions where the imaginary frequency of the transition state was less than 100 cm^−1^ in magnitude, as these typically correspond to conformational changes.

To avoid excessive computational cost, DFT methods had to be used to generate the reaction dataset. The functional chosen for the string method calculations, B97-D3, does not provide accurate activation energies, but was selected due to its low computational cost. However, *ω*B97X-D3 has been shown to yield excellent quantitative barrier heights with a 2.28 kcal mol^−1^ root-mean-square deviation from reference data that is estimated to be more than ten times as accurate as the best density functionals^41^, which makes this data very useful. Therefore, the following analysis was only completed for the *ω*B97X-D3 data.

**Fig. 2:**
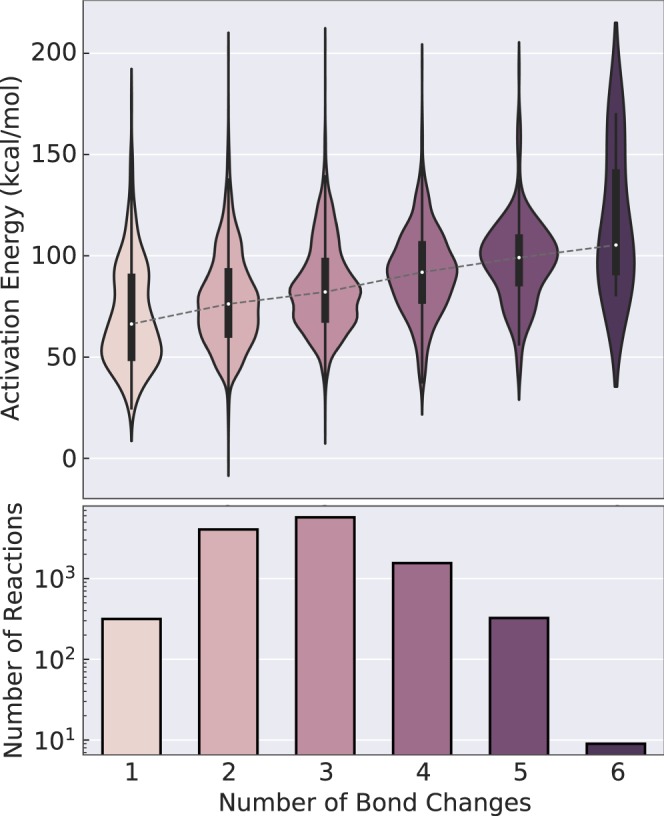
Activation energy distributions. The distribution of activation energies split by the number of bond changes in the *ω*B97X-D3 reactions. Bond changes only consider changes in connectivity between atoms, irrespective of bond order. The distributions are scaled to have equal area.

In order to show that the dataset provides a reasonably diverse set of reactions spanning many different chemistries even though constraints were set on the number of atoms and driving coordinate generation parameters, it is necessary to characterize the types of reactions. Figure 1 already shows that the range of activation energies and enthalpies of formation is very large. Even high-energy reactions involving barriers of up to 200 kcal mol^−1^ are included in the dataset. If the data are used to learn reaction prediction models, including such high-energy paths is important in order to not bias models towards the low-energy regions. Figure 2 shows that even though the driving coordinates were limited to three bond changes, significantly more complex reactions involving more bond changes occur in the dataset. Nonetheless, most elementary reactions predominantly occur with only two or three bond changes. Furthermore, the median activation energy increases with an increasing number of bond changes, which is expected.

**Fig. 3:**
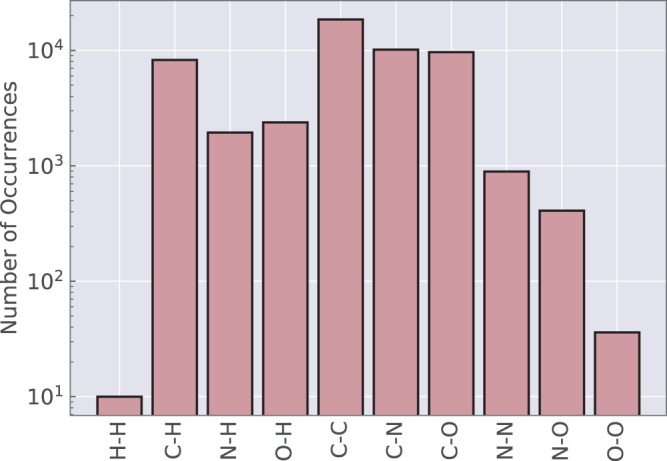
Bond change types. The number of times each type of bond change occurs in the *ω*B97X-D3 reactions. For example, C–N denotes both forming a bond between C and N atoms and breaking a bond between the atoms. This also includes a change in the bond order between the two atoms.

Instead of simply counting the number of bond changes, the reactions can be classified based on the types of bonds that are changed. Figure 3 shows that all combinations of bond changes between H, C, N, and O atoms occur in the dataset with many examples present for all reaction types. H–H changing reactions are the rarest because they only correspond to hydrogen molecule formation.

**Fig. 4:**
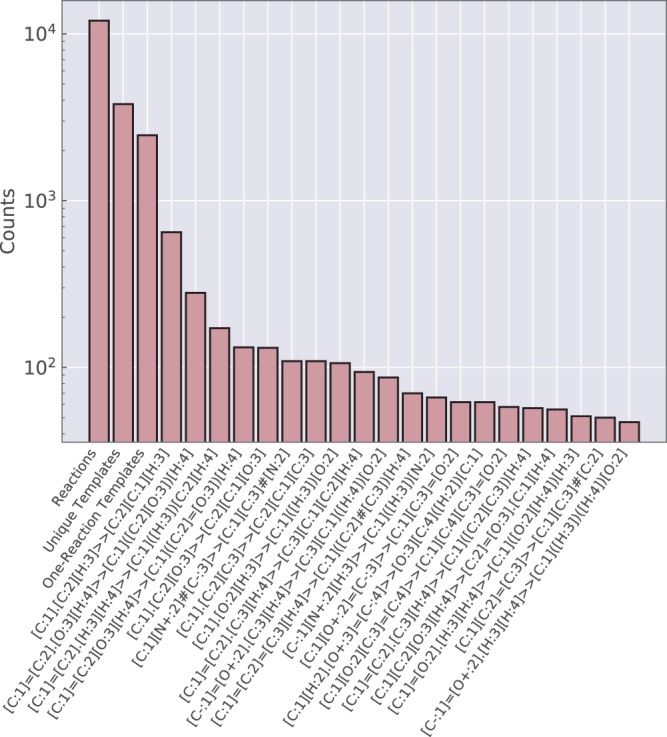
Automatically extracted reaction templates. The reactions were grouped with very general reaction templates that only consider connectivity of atoms in the reactive center, atom identity, charge, aromaticity, and bond type. The top 20 templates are denoted with SMARTS strings^45^.

Lastly, we characterized the reaction diversity by automatically extracting a set of general templates. We only focused on the reactive center by using RDKit’s *GetReactingAtoms* method to isolate atoms changing in the reaction. The molecular fragments in the reactants and products identified as the reactive center were then concatenated together to form the reaction template. In addition to the connectivity of the reacting atoms, the only features considered were atom identity, charge, aromaticity, and bond type. Figure 4 shows the results of this automated extraction. Many templates only have a single reaction example and only the eight most popular templates have more than 100 reaction examples, highlighting the diversity present in the dataset.

## Usage Notes

With the exception of the growing string method code, which is available from the developers of the method^42^, and the Q-Chem quantum chemistry package, all code necessary to reproduce the generated data is available on GitHub^43^. The repository contains several scripts, which should be run in the following order:

- parse_qm9.py: Converts the QM9 data directory^44^, which contains the GDB-9 SMILES along with quantum mechanically derived properties, to a pickled file containing a list of MolData objects, which store the information in QM9 as Python objects.
- make_opt_jobs.py: Performs conformer searches and makes Q-Chem input files for optimization of reactant geometries based on the QM9 SMILES. The geometry optimizations themselves have to be performed with Q-Chem outside of the code, preferably in a massively parallel fashion on a supercomputer.
- create_gsm_jobs.py: Reads the geometry optimization outputs of the reactant optimizations, generates driving coordinates, and writes the files required for the GSM calculations. The GSM code has to be compiled separately^42^. The GSM calculations also have to be run separately and should produce output files with a gsm#.out format, where # corresponds to each reaction path.
- create_prod_optfreq_jobs.py: Reads the string endpoints from the successfully completed GSM calculations and writes the Q-Chem input files for the product optimizations.
- create_ts_optfreq_jobs.py: Extracts the TS geometries from the GSM output files, removes duplicate reactions using the output from the product optimizations, and writes the Q-Chem input files for additional TS optimizations.
- extract_reactions.py: Extracts the unique reactions using the reactant, product, and TS optimization outputs in the form of a comma-separated values file containing SMILES, activation energies, and enthalpies of reaction. Can also write the file path information of all relevant log files to the CSV output, which can be used to copy the log files for every reaction.
- refine_reactants.py: Writes Q-Chem input files for reoptimization of the reactants at the higher level of theory.
- refine_products_and_ts.py: Uses the same method as implemented in extract_reactions.py to extract reactions and write Q-Chem input files for the reoptimization of products and TSs at the higher level of theory. After running the Q-Chem jobs, extract_reactions.py can be run again to extract the high-level reactions.

If desired, the levels of theory and the reaction generation settings can be changed in the config folder.

## Data Availability

The code used to generate the data is freely available on GitHub under the MIT license^43^. Further details on how to use it to generate the data are given in the Usage Notes.
